# Assessing the ecological vulnerability of the upper reaches of the Minjiang River

**DOI:** 10.1371/journal.pone.0181825

**Published:** 2017-07-28

**Authors:** Jifei Zhang, Jian Sun, Baibing Ma, Wenpeng Du

**Affiliations:** 1 Center for Mountain Development Research, Institute of Mountain Hazards and Environment, Chinese Academy of Sciences, Chengdu, China; 2 Synthesis Research Center of Chinese Ecosystem Research Network, Key Laboratory of Ecosystem Network Observation and Modeling, Institute of Geographic Sciences and Natural Resources Research, Chinese Academy of Sciences, Beijing, China; 3 School of Earth Science and Resource, Chang'an University, Xian, China; Institute of Tibetan Plateau Research Chinese Academy of Sciences, CHINA

## Abstract

The upper reaches of the Minjiang River (URMR), located on the eastern edge of the Tibetan Plateau in southwestern China, are an important component of the ecological barrier of the Upper Yangtze River Basin. Climate change and human activities have increased the ecological sensitivity and vulnerability of the region, which may pose a threat to the ecological security of the Yangtze River Basin and have negative impacts on local social and economic development. In this study, we analyzed land use and cover change (LUCC) of the URMR between 2000 and 2010, and found that the total rate of LUCC was less than 0.50% during this period. In addition, net primary production (NPP) was employed to describe the changes in ecosystem sensitivity and vulnerability, and the results demonstrated that slightly and moderately sensitive and vulnerable zones occupied the largest area, distributed mainly in forest, shrub, and grassland ecosystems. However, compared with the period from 2000 to 2005, the ecological sensitivity and vulnerability showed a worsening trend in the period 2005–2010. Exploring the relationship between vulnerability/sensitivity and environmental factors, we found that sensitivity and vulnerability were positively correlated with precipitation (>700 mm) and aridity index (>36 mm/°C). The results highlight that the future ecological sensitivity and vulnerability of URMR should be further investigated, and that the LUCC induced by human activities and climate change have caused alteration of in ecosystem vulnerability.

## 1. Introduction

Climate variability is a natural process [1] and periods of heating and cooling over the history of Earth have been determined [2, 3]; however, compared with the past millennium, a different type of change was observed in the 20^th^ and 21^st^ centuries [4, 5]. The average global temperature increased by 0.065°C per decade, with a total change of 0.85°C from 1880 to 2012 [6]. Climate warming has affected ecosystems in different ways [7], particularly in areas with vulnerable ecosystems, such as the Tibetan Plateau. Meanwhile, intensified human activities and the unsustainable use of natural resources have led to greater ecosystem degradation [8]. To understand how to introduce an appropriate balance between socio-economic and environmental systems, it is important to assess their vulnerability [9].

A widely applied concept in social science with decades of history [10], vulnerability is now increasingly used in ecological studies [11]. Vulnerability is useful indicator for description of relationships between physical, biological, social, and economic systems, as well as policy impacts, and can assist decision-makers in attempts to enhance prosperity by reducing risks or hazards [12]. Despite its cross-disciplinary employment, vulnerability, in its most fundamental sense, is described as the propensity or predisposition of a system, subsystem, or system component to be adversely affected, and comprises sensitivity, or susceptibility to harm, and lack of capacity to cope and adapt in the face of environmental change [13]. In the context of climate change, assessment of ecological vulnerability to continuous human disturbance has attracted a great deal of attention worldwide [14–16]. To develop adaptive activities and build resilience, it is crucial to understand ecological vulnerability from spatial and temporal perspectives [17–22].

For vulnerability assessment of ecosystems, critical ecological processes or major service functions, which are sensitive to external disturbance or vital to the ecosystem, are usually taken as the receptors of external disturbance, the responses of which are applied to assess ecological vulnerability [23]. Net primary production (NPP, usually expressed as g carbon m^−2^ yr^−1^), is highly relevant to ecosystem resilience, waste absorption, and the buffering and regulating abilities of ecosystems, as well as to the services of ecosystems to humans [24]. Ma et al. [25] proposed that the damage to key supporting processes, such as NPP, can induce huge impacts on the earth's environment. Ecosystem dynamics [26], ecosystem sensitivity/vulnerability [27–30], and ecosystem resilience [31, 32] have been explored at local [33,34], regional [35–37], and global scales [38–40]. Previous studies also focused on assessment indicators [41,42], and the growing concerns about erosion of ecosystem services has promoted their spatial representation as a powerful tool for application of ecosystem service methods into land use policies [43]. Ecological vulnerability has been analyzed using a comprehensive index system [44]. However, it is difficult to build systems with multiple-indices to evaluate the degree of fragility of the ecology of large-scale environments. Moreover, the single index system was established on a background of specific geographical conditions, and was defined by distinct, regional, and highly-specific characteristics [45]. Net primary productivity (NPP), the most important index representing the structure and function of an ecosystem, is a key component of the global carbon cycle [46]. It is an important link between the biosphere and the atmosphere, is influenced by water fluxes, nutrient cycles, climate variation, and represents the response of vegetation dynamics to environmental change [47]. Thus, the vulnerability and sensitivity of the URMR can be evaluated via investigation of NPP dynamics.

As the largest tributary of the Upper Yangtze River [48], Minjiang is situated in the Sichuan transition zone from basin, hills, and mountains to plateau. The upper reaches of the Minjiang River (URMR) are an important component of the ecological barrier of the Upper Yangtze River Basin, a key landscape boundary and an ecologically fragile region of China [49]. According to the National Biodiversity Conservation Strategy and Action Plan [50] and the Tibetan Plateau Eco-construction and Environmental Protection Plan [51], the URMR is an important site of biodiversity and an area of convergence of multiple biogeographic divisions in China [52]. Due to rapid economic development and urbanization, land use has significantly changed over recent decades [53, 54], and this may have influenced ecosystems and biodiversity, which is known to affect ecosystem productivity [55]. Some researches have reported landscape change, environmental vulnerability, ecosystem restoration, mammalian diversity, and potential ecosystem service value [49, 53, 56–58] in URMR. However, ecological vulnerability and its heterogeneity resulting from land-use change on a local scale in the URMR have rarely been examined.

Taking the URMR as the study area, this paper aimed to trace the spatial-temporal dynamics of ecological vulnerability, based on NPP, in response to LUCC from 2000 to 2010. Moreover, the biophysical/ecological factors that may influence the vulnerability of the URMR are also discussed. In particular, we explore the relationships between ecological vulnerability/sensitivity and precipitation, temperature, and altitude across the URMR. The results have potential to improve understanding of the responses of the local ecosystem to climatic change and human activity. Our findings could also be useful to inform local decision-making concerning specific protection and maintenance interventions aimed at enhancing sustainable land-use management.

## 2. Materials and methods

### 2.1. Study area

**Fig 1:**
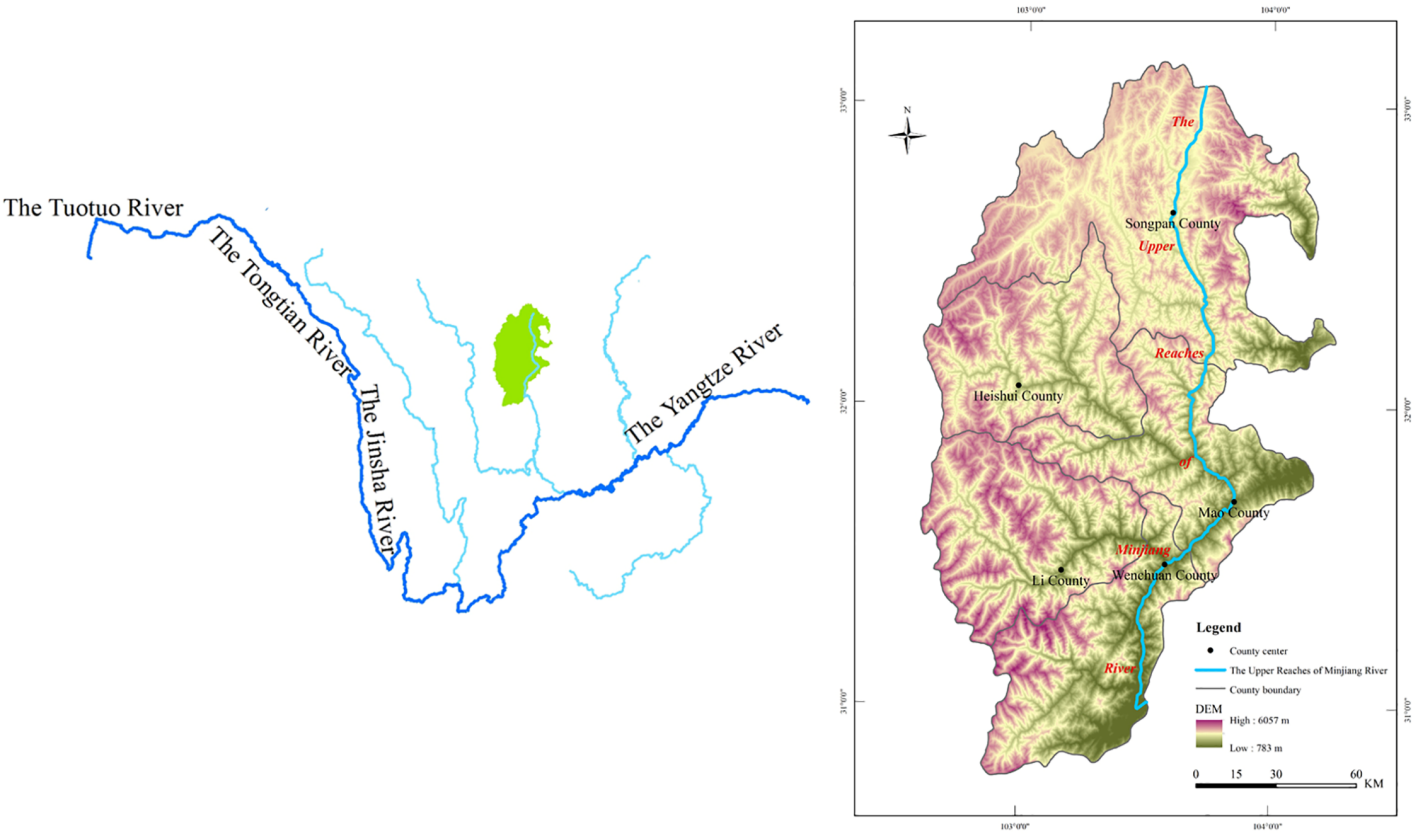
Location of the study area, the URMR.

The Minjiang River is a first tributary of the Yangtze River [58] and a significant area economically and ecologically in southwestern China. It has a drainage basin of approximately 13.6×10^4^km^2^ [59], which accounts for almost 14% of the Upper Yangtze River Basin (Fig 1). It is the main water resource of Sichuan Province and vital for agricultural and industrial production in the region. The Minjiang River Basin is inhabited by many rare species, including the Chinese dove tree and giant panda.

The URMR (31°26′-33°16′N, 102°59′-104°14′E) is 340km in length, covering an area of approximately 24779.80 km^2^, corresponding exactly with the administrative range of five counties: Songpan, Heishui, Mao, Li, and Wenchuan. The URMR has rich forest resources, and the main vegetation is evergreen broad-leaved forest, evergreen coniferous forest, mixed needle leaf, and bushes. Situated on the eastern edge of the Tibetan Plateau (the transition belt from the Sichuan basin to the Tibetan Plateau) the URMR is a typical mountainous region with clear ecological vulnerability and sensitivity. The landform of URMR is dominated by high relief, due to complex mountains and valleys, with elevations from 700 to 6260m and an average elevation difference of >1000m. The URMR is dominated by forest and grassland ecosystem types, which account for 60.24% and 28.80% of the total area, respectively. The northern region has adequate light, low rainfall, and a lack of heat and moisture. In contrast, the southern region has abundant rainfall and seasonal distribution of warm winters and cool summers. There are many nature reserves in the URMR: Wolong, Huanglong, Caopo, Baiyang, and Miyaluo; however, the majority of the region has undergone serious deforestation in recent decades, leading to a huge loss of biodiversity [53]. Moreover, the Ms 8.0 Wenchuan earthquake on May 12, 2008 had a profound impact on local ecosystems and LUCC. In 2014, the total resident population of URMR was 392900 [60], concentrated in the river-valley area, with an urbanization level of 39.14%.

### 2.2. Vulnerability calculation method

The vulnerability of the ecosystem was calculated according to sensitivity and adaptability [61]. The formula is expressed as:

V=S−A (1)

Where *V*, *S*, and *A* represent the vulnerability, sensitivity, and adaptability of the ecosystem, respectively. The vulnerability depends on changes in sensitivity and adaptability of the ecosystem. For a specific ecosystem region, the sensitivity is defined as the response degree of the ecosystem to environmental change. The adaptability is the ability of the ecosystem to maintain and recover its structure in the face of environmental change.

In our study, NPP of vegetation was adopted as the receptor of human disturbance and environmental change. The sensitivity was expressed as the inter-annual fluctuation of ecosystem function. The formula is:

$$S=\sum_{i=1}^{n}|F_{i}-\bar{F}|/\bar{F}$$ (2)

where *F*_*i*_ is the value of NPP in period *i* for ecological function capacity during the study period (2000–2010); $\bar{F}$ is the average value of NPP in the URMR from2000 to 2010; S (sensitivity) indicates the variable rate of ecosystem function, which reflects the degree of dispersion of the average value from 2000 to 2010.

Ecosystem adaptation means the self-regulation mechanism of ecosystems. Presently, the mechanisms of adaption remain unclear at the ecosystem level; however, ecosystem adaptation can be regarded as a measure of maintenance of the system in a relatively stable state. Thus, in a certain period, the trend of variability of an ecosystem is used to measure its deviation from the steady-state, and referred to as ecosystem adaptation [62]. If the change trend of variability is reduced or unchanged, the system tends to be relatively stable, whereas increased variability suggests an unstable system to adapt to environmental change, and may indicate the vulnerability is increasing. Over a specific period, the trend of ecosystem adaptability can be expressed by the slope of the linear fitting trend line of inter-annual variability of the ecosystem functional index. In this study, NPP was used as the ecosystem functional index; hence, the adaptability of the ecosystem was defined by the slope of the linear fitting trend line for inter-annual variability of NPP from 2000 to 2010:

y=ax+b (3)

Where *x* is the inter-annual variability of NPP and *a* is the changing trend of variability (i.e., adaptability), and can be calculated using the following formula [63, 64]:

$$a=\frac{n\sum xy-(\sum x)(\sum y)}{n\sum x_{}^{2}-{\left(\sum x\right)}_{}^{2}}$$ (4)

Where *x* refers to the natural numbers 1, 2, 3…, corresponding to the years from 2000 to 2010, and *y* is identified as the objective variable of NPP.

### 2.3. Data sources

Land use/cover change (LUCC) documents (scale 1:100,000) were retrieved from the National Land Use/Cover Database of China (NLUD-C) (LUCC in China at the end of 2000, 2005, and 2010), developed by Chinese Academy of Sciences [65]. Meanwhile, annual NPP data from 2000 to 2010 was downloaded from the MODIS global data set (MOD17) at an 8 kilometer resolution. The NPP product was the first satellite-driven data set to monitor vegetation productivity based on the NASA Earth Observation System (EOS) program. Climatic data was downloaded from the Meteorology Information Center of the Chinese National Bureau of Meteorology (China Meteorological Data Sharing Service) and included annual mean temperature and annual mean precipitation [60].

## 3. Results

### 3.1. LUCC from 2000 to 2010

**Fig 2:**
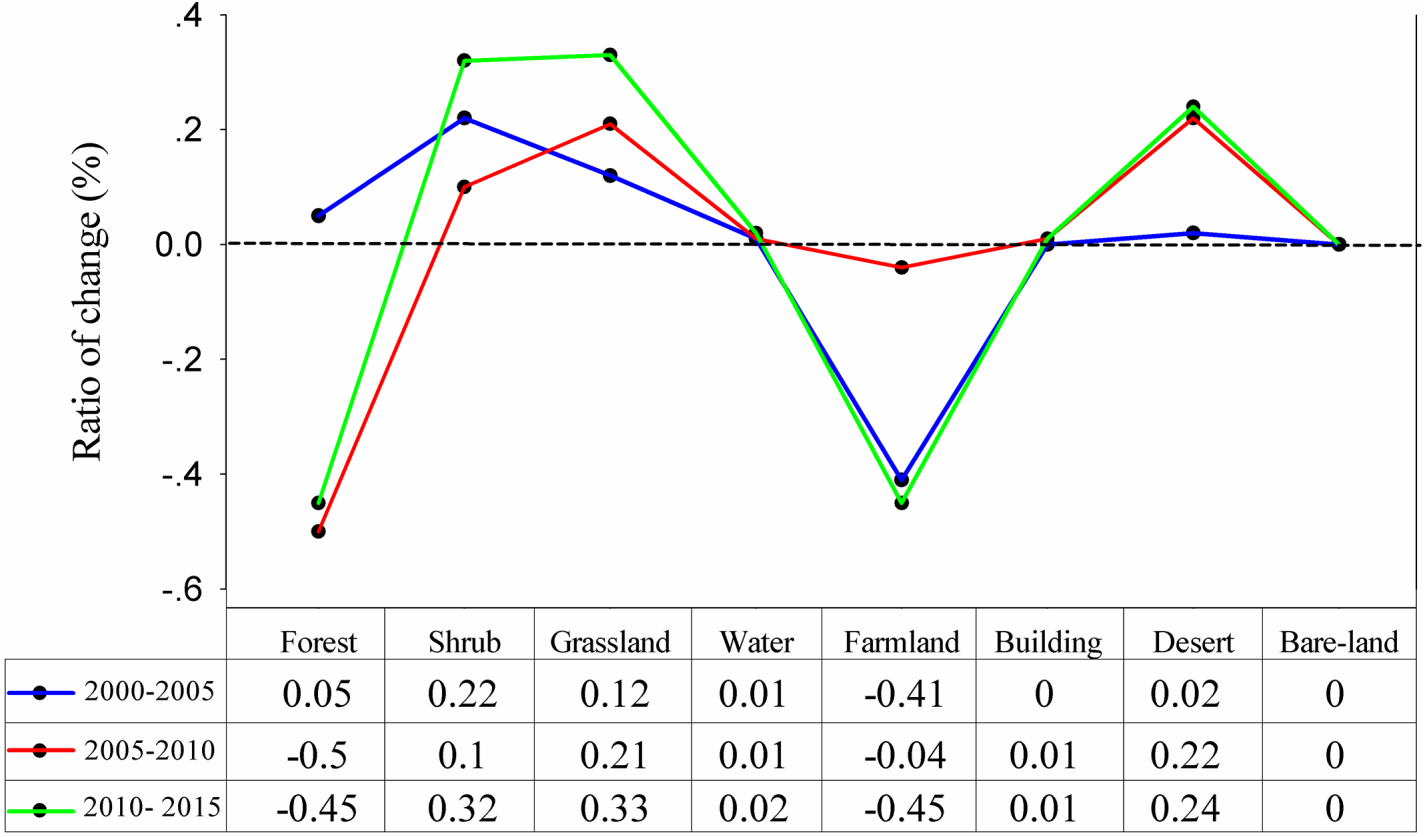
The rate of LUCC in URMR.

LUCC from 2000 to 2010 is summarized in Fig 2 and Table 1. Forest, grassland, and shrub were the main vegetation types, accounting for more than 86% of the study area. Forest increased by 13.09 km^2^ from 2000 to 2005, and decreased by 125.28 km^2^ from 2005 to 2010; the rate of change was -0.45% from 2000 to 2010. The main reason for forest reduction was the Ms 8.0 Wenchuan earthquake, which occurred on May 12, 2008, consistent with the findings of Tian et al. [66] who studied the vegetation damage situation in the URMR during the earthquake and its recovery status in the subsequent two years. Grassland increased by 27.39 km^2^ from 2000 to 2005, and by 52.43 km^2^ from 2005 to 2010; the rate of change was 0.33% from 2000 to 2010. Shrub increased by 54.55 km^2^ from 2000 to 2005, and by 24.75 km^2^ from 2005 to 2010; the rate of change was 0.32% from 2000 to 2010. Overall, the total rate of LUCC was less than 0.50% from 2000 to 2010. Forest and farmland decreased, with bare-land remaining stable and other types of land use increasing.

**Table 1:** Land use types in the URMR in 2000, 2005, and 2010yr.

Land-use type	2000		2005		2010		Percentage change (%)		
	Area (km^2^)	Percentage (%)	Area (km^2^)	Percentage (%)	Area (km^2^)	Percentage (%)	2000– 2005	2005– 2010	2000– 2010
Forest	9020.28	36.40	9033.37	36.45	8908.09	35.95	0.05	-0.50	-0.45
Shrub	5908.11	23.84	5962.66	24.06	5987.41	24.16	0.22	0.10	0.32
Grassland	7137.79	28.80	7165.18	28.92	7217.61	29.13	0.12	0.21	0.33
Water	67.76	0.27	70.27	0.28	71.70	0.29	0.01	0.01	0.02
Farmland	551.81	2.23	450.89	1.82	442.19	1.78	-0.41	-0.04	-0.45
Building	8.04	0.03	8.60	0.03	9.10	0.04	0.00	0.01	0.01
Desert	1775.37	7.16	1778.19	7.18	1833.06	7.40	0.02	0.22	0.24
Bare-land	310.64	1.25	310.64	1.25	310.64	1.25	0.00	0.00	0.00
Total	24779.80	100	24779.80	100	24779.80	100	/	/	/

### 3.2. Sensitivity of ecosystems

**Fig 3:**
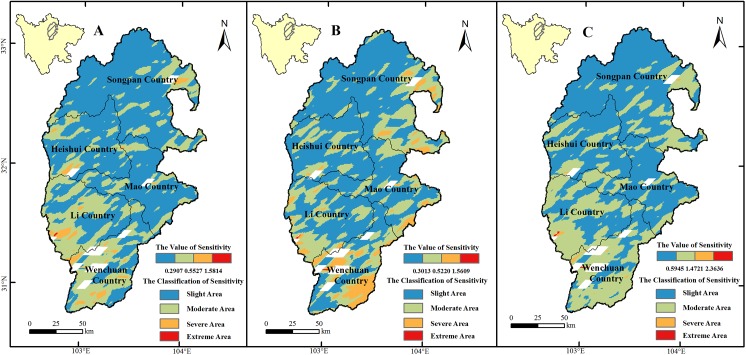
Ecosystem sensitivity of the URMR. The graph A, B and C represent the sensitivity of the URMR during 2000-2005yr, 2005-2010yr, and 2000-2010yr, respectively.

According to cluster analysis, the ecosystem sensitivity was classified into four types (slight, moderate, severe, and extreme) in three different study periods (2000–2005yr, 2005–2010yr, and 2000–2010yr) (Fig 3 and Table 2). The slightly and moderately sensitive zones were the largest in our study area, the severely sensitive zone was small and mainly distributed in the southern area, and there was almost no extremely sensitive zone, suggesting that the overall sensitivity level of the study area was ‘slight’. From 2000 to 2010, the combined area of the slightly and moderately sensitive zones was 24037.25 km^2^, accounting for more than 99% of the study area, while severely and extremely sensitive zones made up less than 1%. Comparing the periods 2005–2010 and 2000–2005, the slightly sensitive zone decreased from 15818.36 km^2^ to 15100.40 km^2^ (change rate, -2.96%), the moderately sensitive zone decreased from 7700.71 km^2^ to 7490.88 km^2^ (change rate, -0.86%), and the severely vulnerable zone increased from 549.58 km^2^ to 1466.71 km^2^ (change rate, 3.81%).

**Table 2:** Changes in area of zones of different levels of sensitivity in the URMR during 2000–2005, 2005–2010, and 2000–2010yr.

Sensitivity Level	2000–2005		2005–2010		2000–2010	
	Area (km^2^)	Percentage (%)	Area (km^2^)	Percentage (%)	Area (km^2^)	Percentage (%)
Slight	15818.36	65.71	15100.40	62.75	14852.52	61.60
Moderate	7700.71	31.99	7490.88	31.13	9184.73	38.10
Severe	549.58	2.28	1466.71	6.09	60.38	0.25
Extreme	5.30	0.02	7.45	0.03	12.23	0.05

During 2000–2010, the main severely and extremely sensitive zones located in desert ecosystem, accounting for more than 40.74% area in the severely sensitive zone and 55.56% area in the extremely sensitive zone. Meanwhile, the slight sensitivity area was distributed in the forest and grassland ecosystems (Table 3).

**Table 3:** Changes in area of zones of different levels of sensitivity in the URMR during 2000–2010 according to land-use types.

Land-use Types	Sensitivity Level							
	Slight		Moderate		Severe		Extreme	
	Area (km^2^)	Percentage	Area (km^2^)	Percentage	Area (km^2^)	Percentage	Area (km^2^)	Percentage
Forest	5083.98	34.42	3658.64	40.23	5.15	7.41	0.00	0.00
Shrub	3891.33	26.35	1933.65	21.26	6.87	9.88	1.72	11.11
Grassland	4723.35	31.98	2246.19	24.70	24.90	35.80	4.29	27.78
Water	40.36	0.27	30.91	0.34	0.86	1.23	0.00	0.00
Farmland	316.84	2.15	120.21	1.32	0.00	0.00	0.00	0.00
Building	5.15	0.03	1.72	0.02	0.00	0.00	0.00	0.00
Desert	659.43	4.46	923.89	10.16	28.33	40.74	8.59	55.56
Bare-land	48.94	0.33	178.60	1.96	3.43	4.94	0.86	5.56

### 3.3. Vulnerability of ecosystems

**Fig 4:**
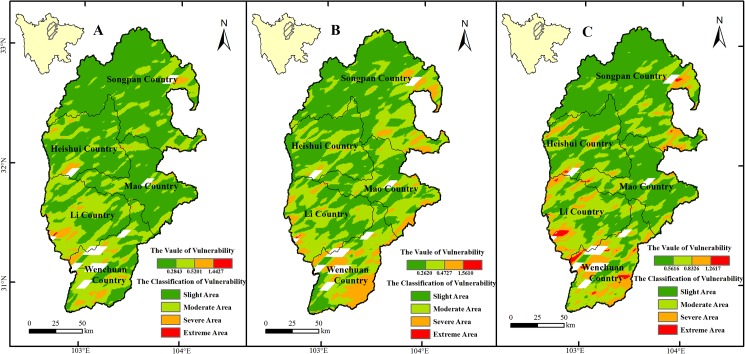
Ecosystem vulnerability of the URMR. The graph A, B and C represent the vulnerability of the URMR during 2000-2005yr, 2005-2010yr, and 2000-2010yr, respectively.

The result showed that the slightly and moderately vulnerable zones were the largest in the study area (Fig 4 and Table 4), and the severely and extremely vulnerable zones were mainly distributed in the south. During 2000–2010, the area of the slightly and moderately vulnerable zones was 21596.39 km^2^, accounting for almost 90% of the total area, while the extremely vulnerable zone made up only 0.85%, and the result indicated that the degree of vulnerability in the study area was ‘slight’. Comparing 2005–2010 with 2000–2005, the slightly vulnerable zone decreased from 15393.17 to 12286.62 km^2^, with a change rate of -12.92%; the moderately vulnerable zone increased from 7891.77to 9664.87 km^2^, with a change rate of 7.36%; and the severely vulnerable zone increased from 772.48 to 2111.96 km^2^, with remarkable change rate of 5.57%; the extreme vulnerable zone was almost invariant. In summary, the moderately and severely vulnerable zones were increased while the slight vulnerability zone was decreased simultaneously, suggesting that the overall vulnerability of the ecosystem deteriorated during the period of 2005 to 2010 compared with 2000 to 2005yr.

**Table 4:** Changes in vulnerability in the URMR during the periods of 2000–2005yr, 2005–2010yr, and 2000–2010yr.

Vulnerability Level	2000–2005		2005–2010		2000–2010	
	Area (km^2^)	Percentage (%)	Area (km^2^)	Percentage (%)	Area (km^2^)	Percentage (%)
Slight	15393.17	63.97	12286.62	51.05	13299.04	55.31
Moderate	7891.77	32.80	9664.87	40.16	8297.35	34.51
Severe	772.47	3.21	2111.96	8.78	2243.43	9.33
Extreme	6.24	0.03	4.06	0.02	203.67	0.85

The slightly, moderately, and severely vulnerable zones were mainly distributed in the forest, shrub, and grassland ecosystems (Table 5), which comprised more than 90%, more than 85%, and more than 80% of the slightly, moderately, and severely vulnerable zones, respectively. The extremely sensitive zone was mainly composed of grassland and desert, which accounted for more than 65%.

**Table 5:** Changes in area of zones of different levels of vulnerability in the URMR during 2000–2010 according to land-use types.

Land-use Types	Vulnerability Level							
	Slight		Moderate		Severe		Extreme	
	Area (km^2^)	Percent-age (%)	Area (km^2^)	Percent-age (%)	Area (km^2^)	Percent-age (%)	Area (km^2^)	Percent-age (%)
Forest	4479.50	33.84	3394.18	41.24	843.18	37.23	30.91	14.34
Shrub	3528.13	26.65	1835.76	22.31	438.76	19.37	30.91	14.34
Grassland	4308.63	32.55	2022.94	24.58	596.75	26.35	70.41	32.67
Water	36.06	0.27	24.90	0.30	9.44	0.42	1.72	0.80
Farmland	285.07	2.15	140.82	1.71	11.16	0.49	0.00	0.00
Building	3.43	0.03	3.43	0.04	0.00	0.00	0.00	0.00
Desert	558.97	4.22	695.49	8.45	293.65	12.96	72.13	33.47
Bare-land	37.78	0.29	112.48	1.37	72.13	3.18	9.44	4.38

### 3.4. Effects of environmental factors on ecosystems

**Fig 5:**
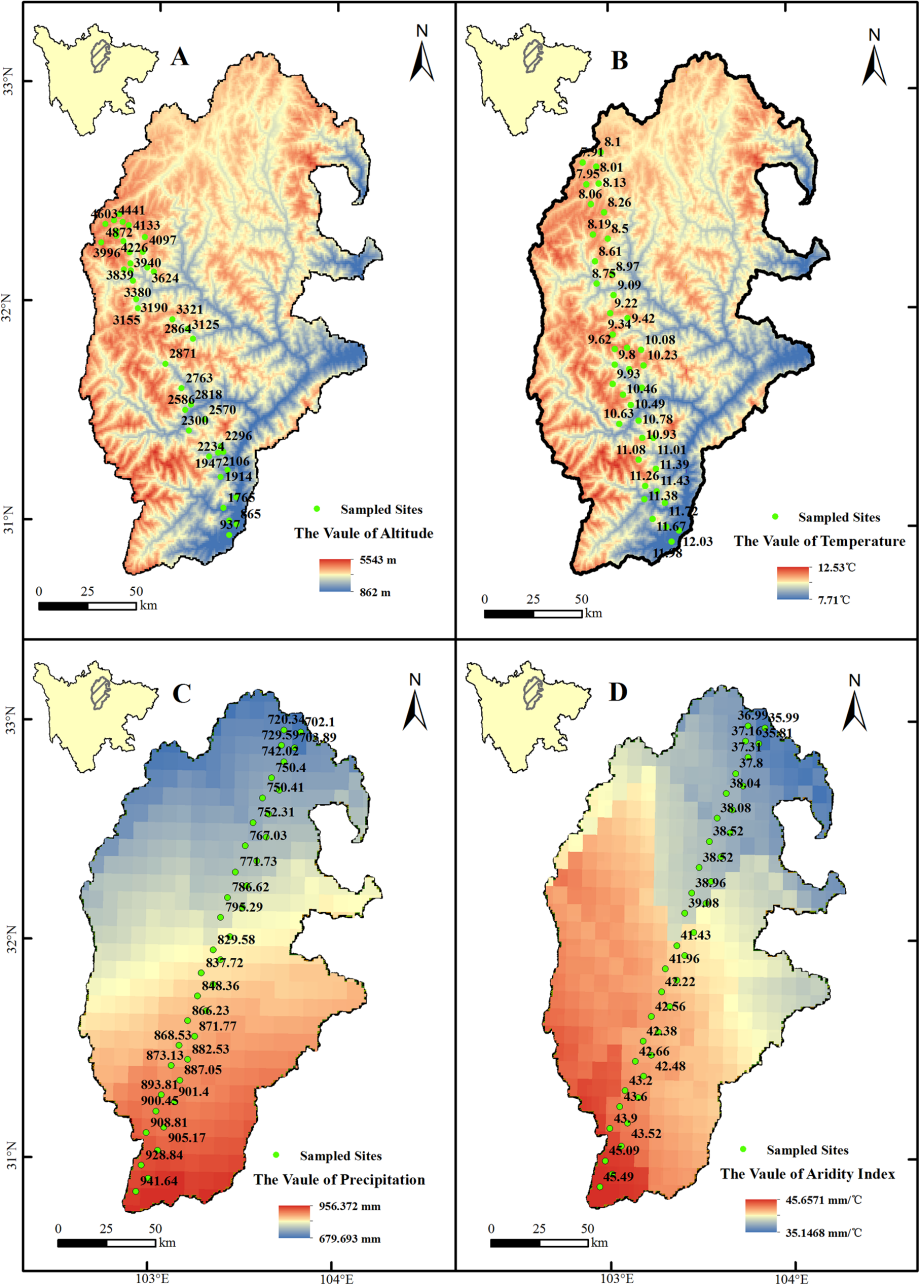
The sampled sites of environmental factors in the URMR. The graph A, B, C and D represent altitude, temperature, precipitation and aridity index gradients, respectively.

**Fig 6:**
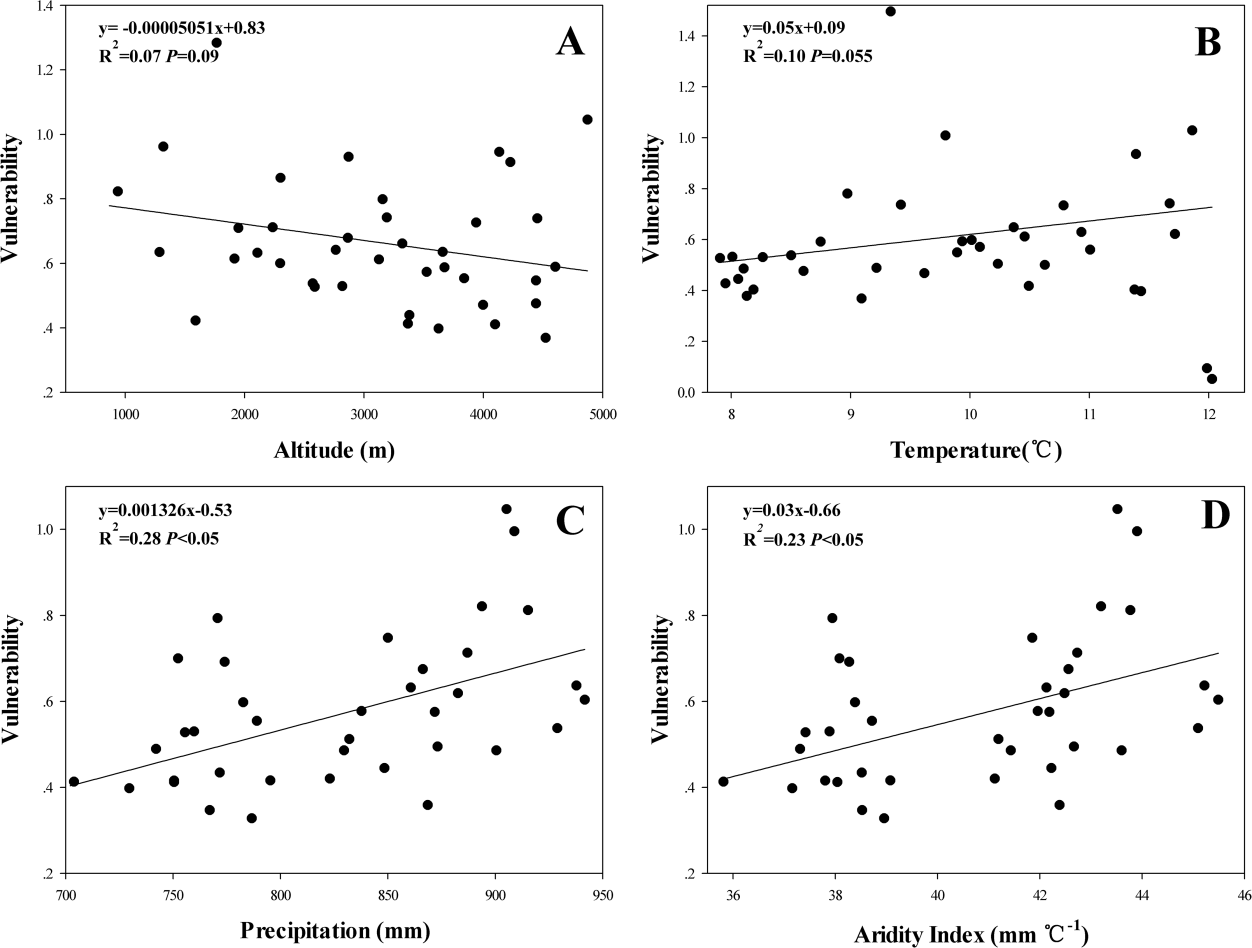
Relationships of environmental factors with vulnerability in the URMR. Graph A, B, C and D represent the effects of altitude, temperature, precipitation and aridity index on the vulnerability, respectively.

To investigate the relationships of sensitivity and vulnerability with altitude, temperature, precipitation, and aridity index, we randomly sampled 40 sites along gradients of altitudinal, temperature, precipitation, and aridity change (Fig 5). Due to sensitivity is closely related with vulnerability, we only explore the relationships of environmental factors with ecosystem vulnerability. There were no significant relationship between vulnerability and altitude (Fig 6A), and temperature also had an insignificant effect on ecosystem vulnerability (*P*>0.05) (Fig 6B). However, the significant positive correlation was identified between precipitation (>700mm) and vulnerability (*R*^2^ = 0.28, *P*<0.05) (Fig 6C). Similarly, a significant positive correlation was found between vulnerability and the aridity index (*R*^2^ = 0.23, *P* < 0.05; >36 mm/°C) (Fig 6D).

## 4. Discussions

### 4.1. Vulnerability and sensitivity of ecosystems

The vulnerability of ecosystems is determined by their degree of exposure, sensitivity, and adaptability. In addition, the degree of exposure reflects external disturbance or stress, which indicates human activity in the area. Sensitivity reflects an area where suffers from the influence of stress, while adaptability is the adaptive capacity of the area experiencing stress [67]. The farmland area and human population are small in URMR (the coefficient of cultivated land is 1.74% and the population density is 16 persons/km^2^); therefore, the degree of exposure is not a major factor for vulnerability. Moreover, artificial ecosystems account for only 2% of the area (Table 1), suggesting that the level of human disturbance is relatively low for the terrestrial ecosystem in the study area. Notably, compared with the period from 2000 through 2005, the area of severe and extreme sensitivity was elevated during 2005–2010 (Table 2). Further study showed that the main land use types resulting in deterioration of sensitivity were farmland and buildings. Urbanization is proceeding worldwide, particularly in China, which is a rapidly developing country. Agricultural land is being transformed into building land during the process of urbanization, which accelerates environmental degradation [68]. Our results demonstrate that the slightly and moderately vulnerable zones were the largest areas in the URMR during the period from 2000 to 2010. Similar results were reported for vulnerability in China based on potential vegetation and climate change [69, 70]. After analyzing the spatial pattern of vulnerability in the URMR, we propose that the vulnerability of Songpan County is low, because it is located on the source of the Minjiang River (a national natural protection zone), and has a small population with low levels of human activity. Wenchuan County is more vulnerable because it is almost entirely located in the dry hot valley and suffers more from mountain hazards (i.e., landslide and debris flow) [71].

LUCC has a considerable effect on global climate change [72], the cycle of geochemical elements [73], soil, water [74], and the structure and function of regional ecology [75], leading to increased ecosystem sensitivity and vulnerability [76]. Human activity also has a considerable influence on the sensitivity and vulnerability of ecosystems [77]. Comparing the beginning with the end of the study period, there was a change of 337.20 km^2^ in land-use and land-cover categories (Table 1), accounting for only 1.36% of the total area. The sensitivity and vulnerability of the study area were almost unchanged from 2000 to 2010, although some regions were more sensitive and vulnerable than others (Figs 3 and 4).

### 4.2. Effects of environmental factors on ecosystems

Vulnerability is defined as the degree or ease with which a system suffers or fails to cope with the effects of climate change [78, 79]. Altitude, temperature, and precipitation also have a great impact on NPP [80, 81]. Within the study area, precipitation increased from north to south, and increased precipitation is associated with an elevated likelihood of soil erosion, landslide, and debris flow [82], which contribute to the increased vulnerability of an ecosystem; hence, there is a positive correlation between vulnerability and precipitation. In general, high productivity lowered the vulnerability of the ecosystem. Temperature also has an impact on productivity, with some studies demonstrating that NPP increases with rising temperature [83, 84]. However, other studies reported that the vegetation respiration rate is accelerated when temperatures increase, which reduces the NPP of ecosystems [85, 86]. The aridity index was calculated from precipitation and temperature, and higher aridity indices were associated with elevated vulnerability(Fig 6D). Furthermore, the terrain in the study area is considerably undulated, with an altitude range of over 1000m. Therefore, the vertical distribution of the climate and vegetation is noticeable in the URMR [87]. The vegetation types changes from temperate forest into dry-valley shrub, subalpine forest, subalpine meadow, and shrub as the altitude increases [88, 89]. Structure and function are important features of ecosystems, and the specific structures of all ecosystems influence their functions as well as their performance [90]. The more complex an ecosystem structure and the more powerful the ecosystem function is, the stronger the ability of the ecosystem to resist interference, and the higher its stability and lower its vulnerability [91, 92]. Hence, gradual weakening of the structure and function of ecosystems results in changes which make it more vulnerable. Nevertheless, the vertical change in vegetation and climate also has a different impact on the vulnerability of ecosystems, and the reason for the correlation between altitude and vulnerability in the study area need to explore in next step.

## 5. Conclusions

In this study, NPP was used to assess ecological vulnerability in the Upper Reaches of the Minjiang River, Eastern Tibetan Plateau, China. Based on the change trend of NPP from 2000 to 2010, the sensitivity and vulnerability of the ecosystems were analyzed. Furthermore, the correlations of vulnerability and sensitivity with environmental factors were explored. We reached the following conclusions:

1. The total changed rate of LUCC was less than 0.50% from 2000 to 2010 in the URMR, with forest and farmland was decreasing, bare-land was stable, and other types were increasing.
2. Assessment of changes in ecosystem sensitivity and vulnerability indicated that the slightly and moderately sensitive/vulnerable zones occupied the largest area of the URMR, and were mainly distributed in forest, shrub, and grassland ecosystems. Furthermore, an overall deteriorating trend was found in ecological sensitivity/vulnerability of the study area during these years.
3. In addition, positive correlations were identified between sensitivity/vulnerability and precipitation (>700mm) and aridity index (>36 mm/°C) in the URMR from 2000 to 2010.
